# Developing Normative Reference Values for Nerve Conduction Studies Using Electrophysiological Parameters in the Bangladeshi Population

**DOI:** 10.1155/nri/2824530

**Published:** 2026-01-31

**Authors:** S. K. Mahbub Alam, Sahariar Hossain Siddik, M. Ferdousi, F. Deeba

**Affiliations:** ^1^ Department of Neurology, Bangladesh Medical University, Dhaka, Bangladesh; ^2^ Nuclear Safety, Security & Safeguard Division, Bangladesh Atomic Energy Regulatory Authority, Dhaka, Bangladesh; ^3^ Department of Physics, Dhaka University of Engineering & Technology, Gazipur, Bangladesh, duet.ac.bd

**Keywords:** nerve conduction study, normal values, normative data, reference values

## Abstract

This study aims to establish normative reference values for nerve conduction studies specific to the Bangladeshi population. Data were collected from 258 healthy subjects, grouped by age and sex. Both motor and sensory nerves of the upper and lower limbs were assessed. Using the ordinary least square (OLS) regression method, it is seen that for the left median motor nerve, the mean distal latency is 3.01 ± 0.34 ms, amplitude 18.05 ± 4.73 μV, and conduction velocity 59.67 ± 6.64 m/s. For the left median sensory nerve, the latency is 2.30 ± 0.25 ms, the amplitude is 60.82 ± 23.95 μV, and the velocity is 55.87 ± 4.48 m/s. The findings of this study were compared with previously published international data, revealing significant differences. These results provide neurophysicians with population‐specific reference values, enhancing diagnostic accuracy, enabling earlier detection of nerve conduction abnormalities, and guiding more targeted and effective treatment strategies for nerve disorders in Bangladesh.

## 1. Introduction

Nerve conduction studies (NCS) are an important diagnostic tool and are widely used to evaluate the condition of the peripheral nerves. It plays a key role in identifying a range of neurological disorders by evaluating how effectively electrical signals travel through the nerves in the body [1, 2]. NCS assess nerve function by measuring latency, amplitude, and nerve conduction velocity (NCV). Abnormal findings in NCS indicate nerve damage or dysfunction and are identified by comparing patient results with established normative or reference values [1, 3]. These normative values are based on data collected from a large healthy population.

Fixed universal reference limits for NCV are not appropriate, as nerve conduction parameters are influenced by demographic and physical factors that vary across different ethnic and geographic populations [4]. Differences in NCVs are clinically significant, as they help distinguish between demyelinating and axonal neuropathies. Slower NCVs suggest demyelination, while reduced amplitudes with preserved velocities indicate axonal loss. Physiological factors such as age, gender, height, and limb temperature influence NCV of normal healthy individuals; for instance, females often show slightly faster velocities, likely due to shorter limb length. Recognizing these variations is essential for establishing normative electrophysiological data [5, 6] as well as for ensuring accurate diagnosis and effective clinical interpretation.

In Western countries, several studies have been done to establish local normative data for the nerves of the upper and lower limbs [7–11]. Due to the lack of locally available published reference data, most neurophysiology departments around the world that are unable to establish their own reference values rely on established Western norms [12]. Using these Western normative values for other populations who have different body types, lifestyles, and environmental conditions can lead to incorrect clinical interpretations.

Recently, a few studies have been published in Asian countries such as China, India, and Pakistan, aiming to develop their own normative reference values [13]. Studies from Pakistan have demonstrated noticeable variations in sensory NCS compared to American and multiethnic populations [13]. Shivji et al. [13] reported that females consistently exhibited higher sensory nerve action potential amplitudes compared to males, most significantly in upper‐limb nerves. Fong et al. [4] demonstrated ethnic and racial differences in their study. These regional studies reinforce the importance of generating normative data for the Bangladeshi population. Although Bangladesh is a small country with a massive population of 175.66 million, to the best of our knowledge, there is no normative data for NCS for the Bangladeshi population. As Bangladeshi people have distinct differences in physical structures, food habits and lifestyles compared to neighboring countries, developing local normative reference values for NCS will optimize the accuracy of diagnosis, management, and prognosis of related neurological diseases.

The aim of this study is to establish normative reference values for NCS that could be applicable to the Bangladeshi population and can be used in neurophysiology departments around the country. We categorize the NCV values in terms of age and sex to make them more relevant. We also compare the normative reference values with other established normative values for different country populations to understand the impact of the region.

## 2. Materials and Methods

A prospective, descriptive study of willing healthy controls was undertaken over a six‐month period (June 2023–December 2023). Written informed consent was obtained from all the participants. This study was approved by the Departmental Technical Committee and the Institutional Review Board (IRB approval number: 983) of Bangladesh Medical University (BMU). All research was performed in accordance with the relevant guidelines and regulations. The sample was recruited from the Neuromuscular Disorder Clinic, inpatient and outpatients of the Department of Neurology, BMU, Dhaka, Bangladesh, after matching the inclusion and exclusion criteria. A total of 258 healthy volunteers without any sensory symptoms, neurological disorders, or medical conditions that could influence peripheral nerve health (such as diabetes, thyroid problems, vitamin B12 deficiency, or cancer, whether treated with chemotherapy or not), agreed to participate in the study. These participants were subjected to neurological exams, as well as measurements of their height and weight. They also underwent NCS, employing the established protocols for evaluating sensory (median, ulnar, and sural) and motor (median, ulnar, peroneal, and tibial) nerve functions.

Data were collected through face‐to‐face interviews using a semistructured questionnaire having selected variables according to objectives. Demographic profile, clinical history, physical examination findings, electrophysiological findings, related laboratory reports, and other related information of each subject were recorded in a data sheet.

The Nihon Kohden Neuropack MEB‐9400 S1 Series EMG/NCV/EP Measuring System was utilized for both NCS and electromyography. For motor and sensory studies, the system’s low‐ and high‐pass filters were adjusted to 2 Hz and 10 kHz and 20 Hz and 3 kHz, respectively. The sweep speed was maintained at 2 milliseconds per division.

Standard techniques were employed to record nerve conduction responses, ensuring supramaximal stimulation and correct electrode placement for accuracy. Data were collected for the various parameters, including onset and peak latencies, amplitude, amplitude drop, conduction velocity, and *F*‐waves. Sensory nerves were tested antidromically. For sensory nerve action potentials (SNAP), amplitude measurements were taken from the peak of the negative potential to the peak of the positive potential. SNAP peak and onset latencies were recorded. For this purpose, the median and ulnar sensory nerves were stimulated at the wrist, and responses were recorded 14 cm away from the index and little fingers, respectively. For palmar response tests, the stimulation distance was set to 8 cm.

For median and ulnar nerve motor testing, the active electrode was placed at the motor points of the abductor pollicis brevis and abductor digiti minimi muscles, respectively. For the tibial, peroneal, and sural nerves in the lower limbs, the active electrodes were positioned over the adductor hallucis and extensor digitorum brevis muscles, respectively. Reference electrodes were placed on the tendons of the respective muscles. Distal stimulation points were set 8 cm away from the active electrode for the motor median, ulnar, and peroneal nerves, 9 cm away for the tibial nerve, and 8–12 cm away for the sural nerve. To conduct NCS, the acceptable limb temperature was ≥ 32°C. If limbs were too cool, hot water bags or heating pads were used to warm the participant to the required temperature.

Statistical analysis was performed using the ordinary least squares (OLS) regression method. Continuous variables were presented in terms of their mean and standard deviation (SD).

## 3. Results

Figures 1–4 show the variation of motor nerve conduction for the upper and lower limbs with age. In Figure 1(a), the regression analysis for the left motor median nerve showed *R* squared = 0.110, adjusted *R* squared = 0.105, intercept coefficient = 66.04, and standard error = 6.283, and in Figure 1(b), *R* squared = 0.093, adjusted *R* squared = 0.088, intercept coefficient = 63.90, and standard error = 4.656 for the right motor median.

FIGURE 1Regression analysis for left and right motor median. (a) NCV for left median versus age. (b) NCV for right median versus age.

**Figure:**
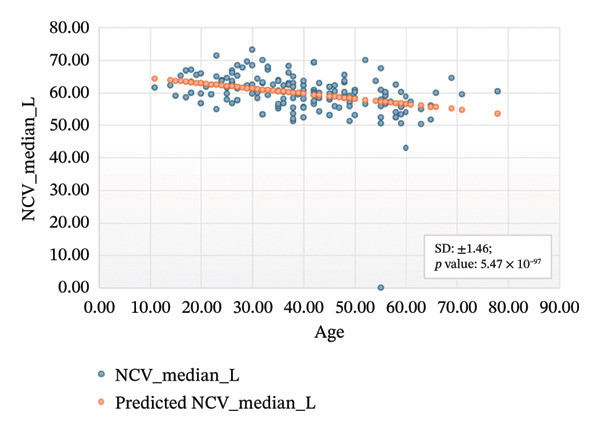
(a)

**Figure:**
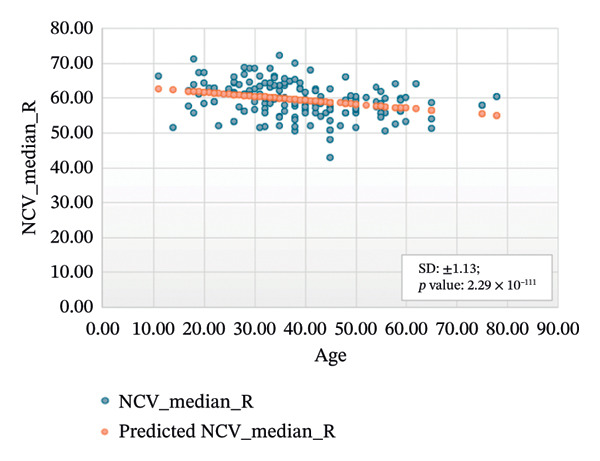
(b)

FIGURE 2Regression analysis for left and right motor ulnar. (a) NCV for left ulnar versus age. (b) NCV for right ulnar versus age.

**Figure:**
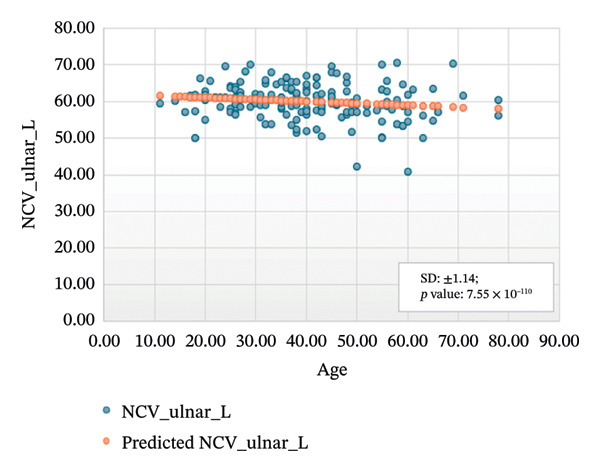
(a)

**Figure:**
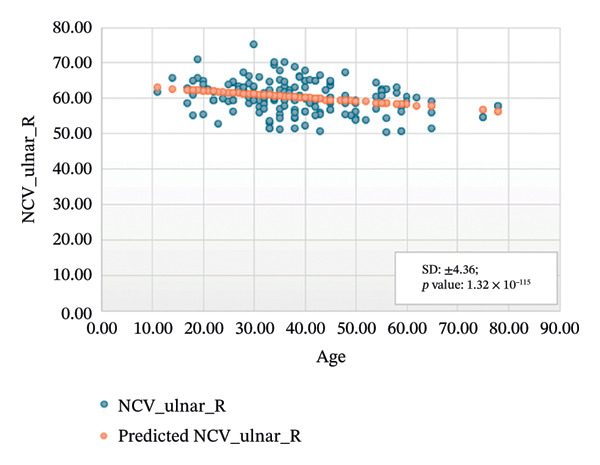
(b)

FIGURE 3Regression analysis for left and right motor tibial. (a) NCV for left tibial versus age. (b) NCV for right tibial versus age.

**Figure:**
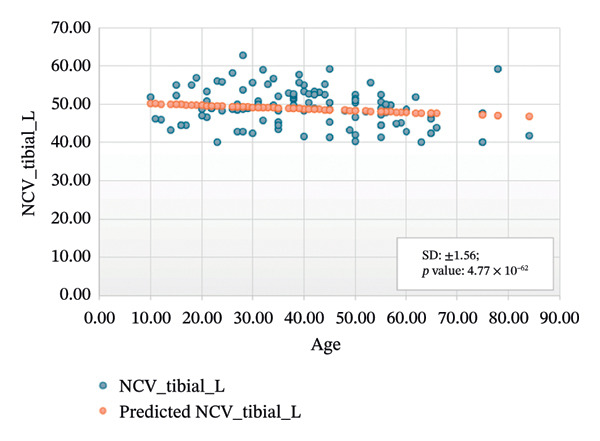
(a)

**Figure:**
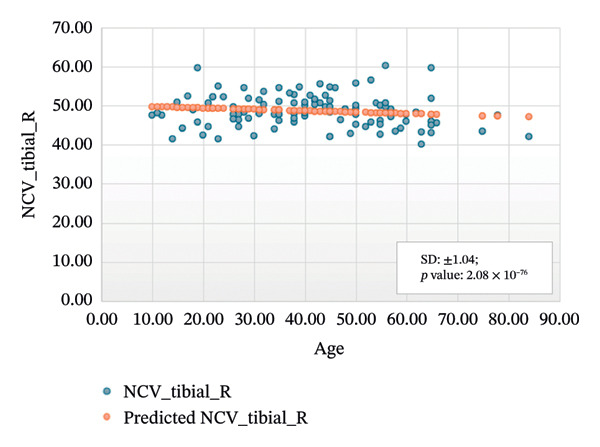
(b)

FIGURE 4Regression analysis for left and right motor peroneal. (a) NCV for left peroneal versus age. (b) NCV for right peroneal versus age.

**Figure:**
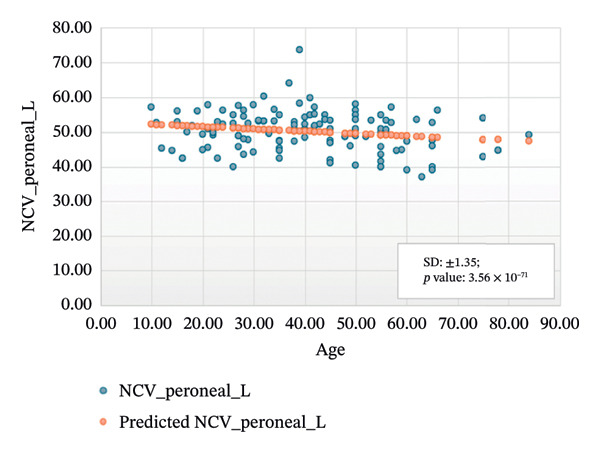
(a)

**Figure:**
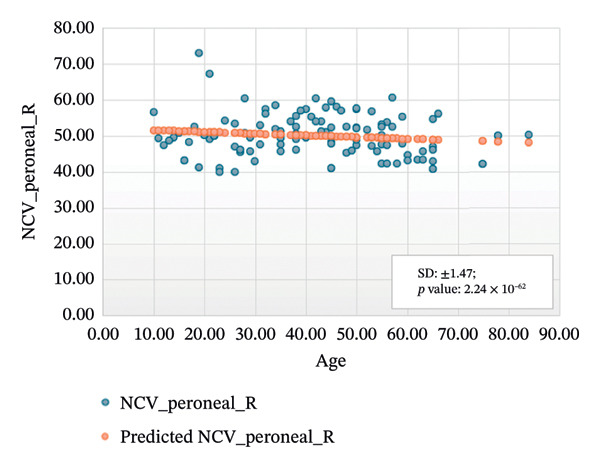
(b)

Figures 2(a) and 2(b) show the analysis results with values *R* squared = 0.022, adjusted *R* squared = 0.016, intercept coefficient = 62.12, standard error = 4.911 and *R* squared = 0.078, adjusted *R* squared = 0.072, intercept coefficient = 63.66, and standard error = 4.364 for the left and right motor ulnar nerve, respectively.

Figure 3(a) presents the analysis results for the left motor tibial, with *R* squared = 0.014, adjusted *R* squared = 0.006, intercept coefficient = 50.60, and standard error = 6.549. Figure 3(b) displays the analysis results for the right motor tibial, with *R* squared = 0.021, adjusted *R* squared = 0.013, intercept coefficient = 50.07, and standard error = 4.096. In Figure 4(a), we find that *R* squared = 0.037, adjusted *R* squared = 0.029, intercept coefficient = 52.74, and standard error = 5.659 for left motor peroneal and in Figure 4(b), we find that *R* squared = 0.0189, adjusted *R* squared = 0.010, intercept coefficient = 52.10, and standard error = 5.813 for right motor peroneal. The correlation of age on conduction velocities of motor median, ulnar, tibial, and peroneal nerve shows that NCV decreases with increasing age in Figures 1–4, respectively.

Figures 5–7 show the variation of sensory nerve conduction for the upper and lower limbs with age. Figure 5(a) presents the results for the left sensory median, with *R* squared = 0.025, adjusted *R* squared = 0.019, intercept coefficient = 57.93, and standard error = 4.421. Figure 5(b) displays the results for the right sensory median, with *R* squared = 0.023, adjusted *R* squared = 0.017, intercept coefficient = 58.20, and standard error = 3.833. Figure 6(a) presents the regression analysis findings for the left sensory ulnar, with *R* squared = 0.009, adjusted *R* squared = 0.004, intercept coefficient = 57.88, and standard error = 6.846. Figure 6(b) presents the analysis findings for the right sensory ulnar, with *R* squared = 0.004, adjusted *R* squared = −0.003, intercept coefficient = 59.11 and standard error = 4.853. The NCV decreases with increasing age for both the left and right sensory ulnar. In Figure 7(a), we find that *R* squared = 7.66 × 10^−6^, adjusted *R* squared = −0.008, intercept coefficient = 50.92, and standard error = 8.636 for left sensory sural. In Figure 7(b), we find that *R* squared = 0.0194, adjusted *R* squared = 0.010, intercept coefficient = 48.62, and standard error = 5.914 for right sensory sural. It is seen that NCV decreases with increasing age for sensory median, ulnar, and sural nerve in Figures 5–7, respectively. We find a significant decrease in NCV with age in the motor nerve conduction compared to the sensory nerve.

FIGURE 5Regression analysis for left and right sensory median. (a) NCV for left median versus age. (b) NCV for right median versus age.

**Figure:**
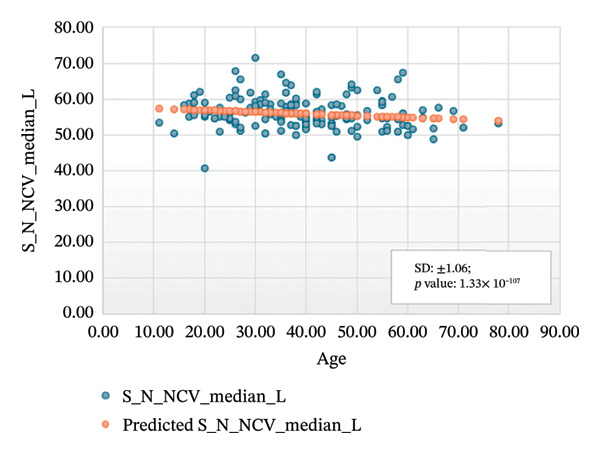
(a)

**Figure:**
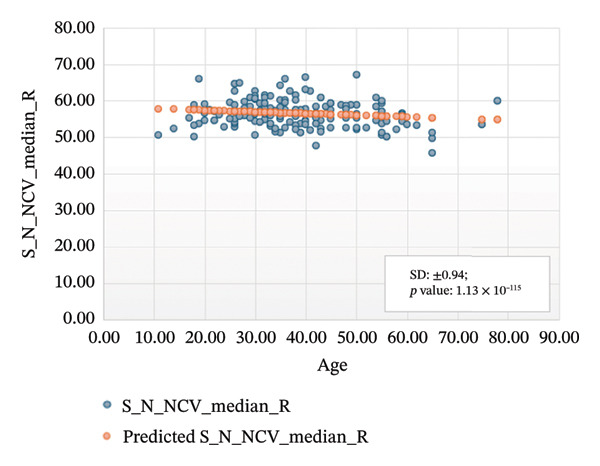
(b)

FIGURE 6Regression analysis for left and right sensory ulnar. (a) NCV for left ulnar versus age. (b) NCV for right ulnar versus age.

**Figure:**
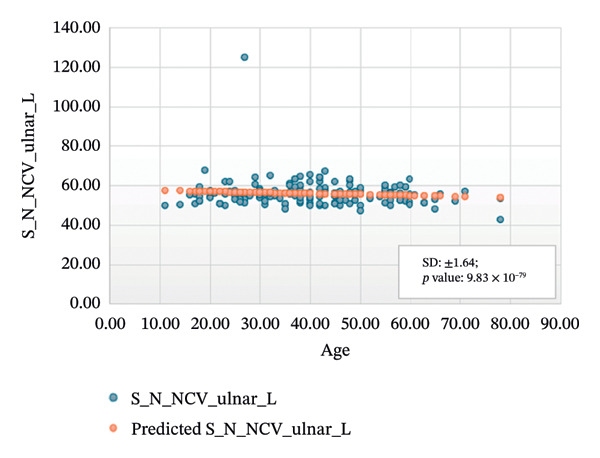
(a)

**Figure:**
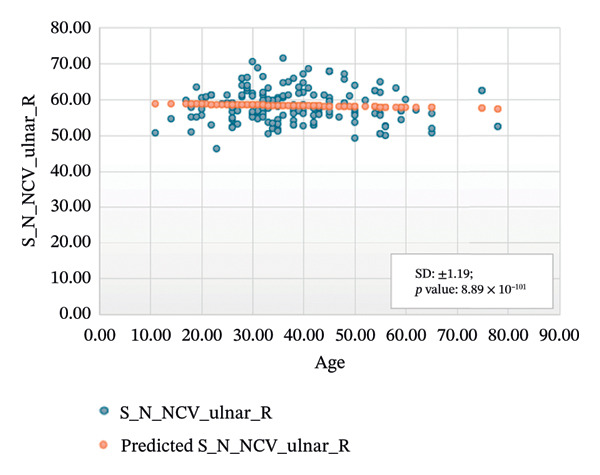
(b)

FIGURE 7Regression analysis for left and right sensory sural. (a) NCV for left sural versus age. (b) NCV for right sural versus age.

**Figure:**
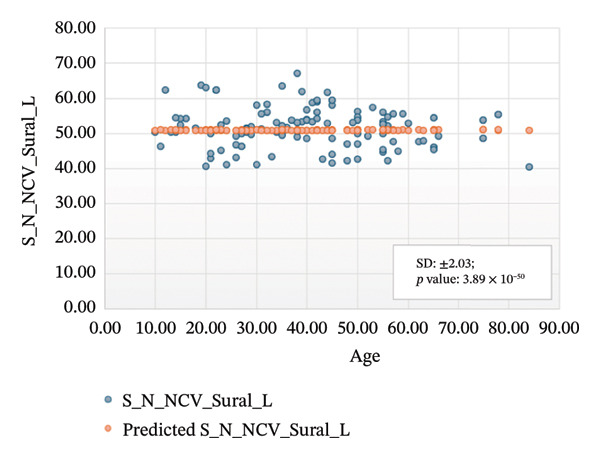
(a)

**Figure:**
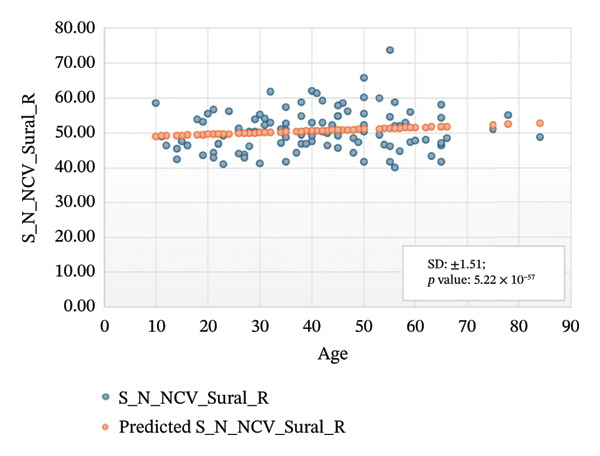
(b)

Motor NCS findings of the upper and lower limbs are summarized in Tables 1–4. For the left median and ulnar motor nerves, data were collected from 173 healthy volunteers (101 females and 72 males), and for the right median and ulnar motor nerves, from 170 healthy volunteers (97 females and 73 males). Participants were grouped into the following age categories: 10–30, 31–60, and over 60 years. Latency, amplitude, and NCV values for median and ulnar motor nerves are detailed in Tables 1 and 2, respectively. The results show that mean latency slightly increases with age, while mean amplitude and NCV slightly decrease with age. Additionally, the average NCV of the median and ulnar nerves is slightly faster in females compared to males.

**TABLE 1:** Motor nerve conduction study findings for median grouped by age.

Motor parameters	Latency ± SD (ms)		Amplitude ± SD (μV)		NCV ± SD (m/s)	
	Left	Right	Left	Right	Left	Right
Mean (total)	3.01 ± 0.34	3.03 ± 0.43	18.05 ± 4.73	17.96 ± 4.93	59.67 ± 6.64	59.45 ± 4.88
Mean (M)	3.18 ± 0.33	3.1 ± 0.40	17.37 ± 4.83	17.0 ± 5.15	58.71 ± 4.54	58.5 ± 4.53
Mean (F)	2.89 ± 0.30	3.0 ± 0.44	18.54 ± 4.62	18.7 ± 4.67	60.36 ± 7.75	60.1 ± 5.04
Mean (10–30 years)	2.94 ± 0.33	2.92 ± 0.43	19.77 ± 4.94	19.34 ± 4.93	62.6 ± 6.64	61.51 ± 4.88
Mean (31–60 years)	3.02 ± 0.35	3.05 ± 0.39	17.52 ± 4.59	17.55 ± 4.77	58.64 ± 7.29	58.72 ± 4.84
Mean (> 60 years)	3.24 ± 0.16	3.45 ± 1.05	15.89 ± 2.92	15.93 ± 3.30	57.47 ± 4.28	58.10 ± 3.99

Abbreviations: F, female; M, male; NCV, nerve conduction velocity; SD, standard deviation.

**TABLE 2:** Motor nerve conduction study findings for ulnar grouped by age.

Motor parameters	Latency ± SD (ms)		Amplitude ± SD (μV)		NCV ± SD (m/s)	
	Left	Right	Left	Right	Left	Right
Mean (total)	2.25 ± 0.30	2.21 ± 0.37	14.11 ± 3.17	14.58 ± 3.41	60.02 ± 4.95	59.89 ± 4.53
Mean (M)	2.46 ± 0.30	2.4 ± 0.32	14.17 ± 3.07	14.1 ± 3.06	58.13 ± 4.90	57.7 ± 4.31
Mean (F)	2.10 ± 0.18	2.1 ± 0.36	14.07 ± 3.25	14.9 ± 3.62	61.37 ± 4.55	61.5 ± 3.98
Mean (10–30 years)	2.169 ± 0.29	2.12 ± 0.37	14.74 ± 3.17	15.25 ± 3.41	60.87 ± 4.95	61.64 ± 4.53
Mean (31–60 years)	2.25 ± 0.26	2.24 ± 0.39	13.93 ± 3.17	14.35 ± 3.36	59.73 ± 5.26	59.45 ± 4.62
Mean (> 60 years)	2.55 ± 0.41	2.35 ± 0.45	13.17 ± 1.82	14.07 ± 2.12	59.29 ± 5.70	56.15 ± 2.87

Abbreviations: F, female; M, male; NCV, nerve conduction velocity; SD, standard deviation.

**TABLE 3:** Motor nerve conduction study findings for tibial grouped by age.

Motor parameters	Latency ± SD (ms)		Amplitude ± SD (μV)		NCV ± SD (m/s)	
	Left	Right	Left	Right	Left	Right
Mean (total)	3.61 ± 0.64	3.75 ± 0.82	18.70 ± 5.77	19.12 ± 7.33	48.71 ± 6.57	48.57 ± 4.12
Mean (M)	3.68 ± 0.66	3.81 ± 0.83	19.00 ± 5.87	19.41 ± 7.86	48.19 ± 7.39	48.39 ± 3.62
Mean (F)	3.51 ± 0.59	3.66 ± 0.83	18.27 ± 5.65	18.64 ± 6.42	49.47 ± 5.12	48.86 4.86
Mean (10–30 years)	3.64 ± 0.63	3.80 ± 0.99	21.46 ± 6.69	21.53 ± 6.75	49.57 ± 5.00	48.39 ± 4.26
Mean (31–60 years)	3.55 ± 0.62	3.69 ± 0.68	17.73 ± 4.82	18.92 ± 7.36	48.74 ± 7.29	49.21 ± 3.74
Mean (> 60 years)	3.87 ± 0.68	3.90 ± 0.98	16.38 ± 5.26	14.85 ± 6.63	46.41 ± 6.23	46.13 ± 4.75

Abbreviations: F, female; M, male; NCV, nerve conduction velocity; SD, standard deviation.

**TABLE 4:** Motor nerve conduction study findings for peroneal grouped by age.

Motor parameters	Latency ± SD (ms)		Amplitude ± SD (μV)		NCV ± SD (m/s)	
	Left	Right	Left	Right	Left	Right
Mean (total)	3.65 ± 0.72	3.56 ± 0.59	7.50 ± 3.25	7.71 ± 3.20	50.02 ± 5.74	50.11 ± 5.84
Mean (M)	3.83 ± 0.78	3.70 ± 0.71	7.80 ± 3.65	7.91 ± 3.14	48.99 ± 5.09	49.37 ± 5.37
Mean (F)	3.40 ± 0.52	3.34 ± 0.57	7.08 ± 2.54	7.39 ± 3.31	51.51 ± 6.34	51.31 ± 6.42
Mean (10–30 years)	3.78 ± 1.00	3.66 ± 0.88	8.67 ± 3.27	8.29 ± 3.84	50.03 ± 5.04	49.54 ± 7.16
Mean (31–60 years)	3.62 ± 0.56	3.48 ± 0.56	7.20 ± 3.30	8.07 ± 2.84	50.67 ± 5.89	51.22 ± 4.96
Mean (> 60 years)	3.49 ± 0.45	3.73 ± 0.63	5.99 ± 1.86	4.89 ± 1.43	46.90 ± 6.06	46.43 ± 4.90

Abbreviations: F, female; M, male; NCV, nerve conduction velocity; SD, standard deviation.

125 healthy volunteers (51 female and 74 male) participated in the study focusing on the left tibial and peroneal nerves, while 113 healthy volunteers (43 female and 70 male) were included in the examination of the right tibial and peroneal nerves. Tables 3 and 4 summarize the latency, amplitude, and NCV for the tibial and peroneal nerves, respectively. The changes in latency and NCV with age are not consistent for the tibial and peroneal nerves. However, the average NCV of the peroneal nerve was found to be slightly faster in females.

For the left median and ulnar sensory nerves study, 167 healthy volunteers (98 female and 69 male) and for the right median and ulnar sensory nerves, 171 healthy volunteers (97 female and 74 male) took part in the experiment. Participants were grouped into the following age categories of 10–30, 31–60, and > 60. Latency, amplitude, and NCV for median and ulnar sensory nerve conductions are presented in Tables 5 and 6. For the sensory median nerve, a slight increase in mean latency with age was observed, accompanied by a slight decrease in mean amplitude and NCV. The changes in latency, amplitude, and NCV with age are not consistent for the sensory ulnar nerve. Additionally, it is found that the sensory average amplitude of the median and ulnar nerves is significantly higher in females.

**TABLE 5:** Sensory nerve conduction study findings for median grouped by age.

Sensory parameters	Latency ± SD (ms)		Amplitude ± SD (μV)		NCV ± SD (m/s)	
	Left	Right	Left	Right	Left	Right
Mean (total)	2.30 ± 0.25	2.33 ± 0.29	60.82 ± 23.95	58.20 ± 21.74	55.87 ± 4.48	54.80 ± 10.27
Mean (M)	2.38 ± 0.25	2.43 ± 0.22	51.27 ± 20.73	48.46 ± 15.44	56.40 ± 4.22	54.17 ± 11.85
Mean (F)	2.25 ± 0.23	2.24 ± 0.31	67.54 ± 23.87	65.62 ± 22.95	55.50 ± 7.26	55.28 ± 8.91
Mean (10–30 years)	2.24 ± 0.27	2.22 ± 0.22	78.55 ± 25.51	68.37 ± 24.85	56.89 ± 9.91	54.75 ± 12.03
Mean (31–60 years)	2.32 ± 0.24	2.36 ± 0.31	55.24 ± 20.00	54.64 ± 18.72	55.68 ± 4.30	54.93 ± 9.78
Mean (> 60 years)	2.42 ± 0.13	2.48 ± 0.26	45.84 ± 17.70	46.59 ± 22.21	53.67 ± 2.78	53.24 ± 4.82

Abbreviations: F, female; M, male; NCV, nerve conduction velocity; SD, standard deviation.

**TABLE 6:** Sensory nerve conduction study findings for ulnar grouped by age.

Sensory parameters	Latency ± SD (ms)		Amplitude ± SD (μV)		NCV ± SD (m/s)	
	Left	Right	Left	Right	Left	Right
Mean (total)	2.05 ± 0.24	1.96 ± 0.29	47.82 ± 19.46	46.63 ± 19.55	55.89 ± 6.86	57.93 ± 6.53
Mean (M)	2.20 ± 0.21	2.10 ± 0.34	39.46 ± 16.45	36.35 ± 14.04	55.13 ± 9.41	55.55 ± 7.75
Mean (F)	1.94 ± 0.19	1.85 ± 0.17	53.70 ± 19.33	54.47 ± 19.58	56.42 ± 4.23	59.74 ± 4.72
Mean (10–30 years)	2.05 ± 0.23	1.91 ± 0.21	59.28 ± 22.24	56.36 ± 21.16	56.55 ± 11.28	57.13 ± 9.54
Mean (31–60 years)	2.04 ± 0.23	1.97 ± 0.31	44.14 ± 16.30	42.28 ± 17.53	55.98 ± 4.24	58.42 ± 4.83
Mean (> 60 years)	2.15 ± 0.30	2.04 ± 0.28	38.90 ± 20.39	49.08 ± 16.62	51.92 ± 4.16	55.69 ± 4.72

Abbreviations: F, female; M, male; NCV, nerve conduction velocity; SD, standard deviation.

For the study of the left sural sensory nerve, a total of 125 healthy volunteers (52 female and 73 male) and for the right sural sensory nerve, 109 healthy volunteers (43 female and 66 male) participated in the experiment. Participants were categorized into age groups of 10–30, 31–60, and over 60. The key findings are detailed in Table 7. It is found that the mean latency and mean amplitude decrease with age for the sural sensory nerve.

**TABLE 7:** Sensory nerve conduction study findings for sural grouped by age.

Sensory parameters	Latency ± SD (ms)		Amplitude ± SD (μV)		NCV ± SD (m/s)	
	Left	Right	Left	Right	Left	Right
Mean (total)	2.38 ± 0.44	2.63 ± 1.47	23.98 ± 15.51	22.56 ± 13.43	50.98 ± 8.60	50.65 ± 5.94
Mean (M)	2.41 ± 0.48	2.84 ± 1.85	24.15 ± 16.21	21.94 ± 13.71	49.84 ± 10.20	49.92 ± 6.31
Mean (F)	2.29 ± 0.34	2.31 ± 0.34	23.74 ± 14.63	23.51 ± 13.09	52.58 ± 5.33	51.77 ± 5.20
Mean (10–30 years)	2.46 ± 0.45	2.57 ± 0.52	30.87 ± 19.04	27.32 ± 17.01	48.76 ± 8.60	48.29 ± 5.94
Mean (31–60 years)	2.35 ± 0.43	2.71 ± 1.90	22.16 ± 13.28	21.12 ± 11.51	52.44 ± 5.32	52.12 ± 6.29
Mean (> 60 years)	2.32 ± 0.46	2.39 ± 0.50	14.64 ± 4.81	17.71 ± 8.41	49.82 ± 4.48	49.67 ± 4.72

Abbreviations: F, female; M, male; NCV, nerve conduction velocity; SD, standard deviation.

The reference values for motor and sensory nerve data from the current study are compared with previously published data [4, 13, 14] in Tables 8–10. The reference limits in Tables 9 and 10 were defined using the 95th percentile for distal latencies and the 5th percentile for amplitudes and conduction velocities.

**TABLE 8:** Comparison of motor and sensory nerve data from the current study with previously published data [4, 13] from other regions of the world.

Parameters	Present study	Chinese	Indian	Malay	Pakistan	USA
Motor median NCV	59.56 ± 5.83	52 ± 2.0	51 ± 2.0	53 ± 2.0	58.7 ± 4.3	48–73
Motor ulnar NCV	59.96 ± 4.74	53 ± 2.0	51 ± 2.0	56 ± 2.0	61.3 ± 3.9	46–71
Motor tibial NCV	48.64 ± 5.53	41 ± 2.0	38 ± 2.0	41 ± 2.0	48.6 ± 4.1	40–60
Motor peroneal NCV	50.07 ± 5.78				50.7 ± 3.9	39–58
Sensory median NCV	55.34 ± 7.97	48 ± 2.0	48 ± 2.0	46 ± 2.0	60.4 ± 4.5	51–71
Sensory ulnar NCV	56.91 ± 6.76	48 ± 2.0	49 ± 2.0	47 ± 2.0	60.0 ± 5.6	49–77
Sensory sural NCV	50.815 ± 7.47	42 ± 2.0	40 ± 2.0	41 ± 2.0	57 ± 6.3	40–58

Abbreviation: NCV, nerve conduction velocity.

**TABLE 9:** Comparison of motor nerve data from the current study with previously published data [4, 14].

Sensory	Present study Reference limits	Fong et al. [4] Reference limits	Preston and Shapiro [14] Reference limits
Median nerve			
Distal motor latency	3.8	4.1	≤ 4.4
Motor nerve conduction velocity	52	52	≥ 49
CMAP amplitude	6.4	7.4	≥ 4.0
Ulnar nerve			
Distal motor latency	3.3	3.0	≤ 3.3
Motor nerve conduction velocity	52	53	≥ 49
CMAP amplitude	6.5	7.0	≥ 6.0
Tibial nerve			
Distal motor latency	4.8	4.1	≤ 5.8
Motor nerve conduction velocity	44	40	≥ 41
CMAP amplitude	6.3	7.5	≥ 4.0
Peroneal nerve			
Distal motor latency	4.3	4.2	≤ 6.5
Motor nerve conduction velocity	44	44	≥ 44
CMAP amplitude	3.1	3.0	≥ 2.0

Abbreviation: CMAP, compound muscle action potential.

**TABLE 10:** Comparison of sensory nerve data from the current study with previously published data [4, 14].

Sensory	Present study Reference limits	Fong et al. [4] Reference limits	Preston and Shapiro [14] Reference limits
Median nerve			
Distal sensory latency	3.2		≤ 3.5
Sensory nerve conduction velocity	50	47	≥ 50
SNAP amplitude	23	7	≥ 20
Ulnar nerve			
Distal sensory latency	2.7		≤ 3.1
Sensory nerve conduction velocity	50	48	≥ 50
SNAP amplitude	23	6	≥ 17
Sural nerve			
Distal sensory latency	3.2		≤ 4.4
Sensory nerve conduction velocity	49	41	≥ 40
SNAP amplitude	8	7	≥ 6.0

Abbreviation: SNAP, sensory nerve action potential.

## 4. Summary and Discussion

NCS are an important tool for evaluating peripheral nervous system disorders. In this study, we established reference values for NCS of commonly tested nerves in healthy Bangladeshi volunteers and compared them with existing normative data. The main results are as follows:

1. Latency is shorter in females both for motor and sensory nerves (Tables 1–7). These findings are consistent with earlier work showing that anatomical and physiological differences such as body size, shorter limb contribute to the observed differences in females [7, 15, 16].
2. The mean latency increases with age in the motor median and ulnar nerves, as well as in the sensory median nerve [14, 17]. This study found a slight decrease in the mean latency of the sural nerve with increasing age, which is not consistent with usual observations, as aging is typically associated with prolongation of latency due to axonal loss and distal nerve degeneration. One possible explanation for this finding is measurement variability related to low‐amplitude responses in older participants, which may make the precise determination of onset latency more challenging. In such cases, the peak latency might appear earlier if the onset is difficult to identify. Additionally, selection bias may have influenced the result, as our older participants were all clinically healthy and may not represent the broader aging population with typical degenerative changes.
3. The amplitude decreases with increasing age for both the motor and sensory nerves. The amplitude drop is significant for sensory nerves with increasing age. This could be explained by the natural loss of axons associated with aging [4, 17].
4. The amplitude of the sensory median and ulnar nerves differs significantly between males and females. The amplitude for median: male = 51.27 ± 20.73 (left) and 48.46 ± 15.44 (right), female = 67.54 ± 23.87 (left) and 65.62 ± 22.95 (right) and amplitude for ulnar: male = 39.46 ± 16.45 (left) and 36.35 ± 14.04 (right), female = 53.70 ± 19.33 (left) and 54.47 ± 19.58 (right). The results are similar to those observed in previous studies [4, 15, 18]. Bolton and Carter [16] suggested that this discrepancy may be attributed to differences in finger circumference.
5. The NCV is faster in females for the motor nerves (Tables 1–4) as well as for the sensory nerves (Tables 5–7). This difference may be attributed to physiological differences such as shorter limb length and consequently reduced signal travel distance, resulting in quicker conduction times. Hormonal influences may also contribute to these differences.
6. From regression analysis (Figures 1–7) it is observed that the NCV decreases with increasing age both for the motor and sensory nerves [19]. These changes may be due to the gradual loss of myelinated nerve fibers, thinning or degeneration of the myelin sheath, and reduced ability of nerves to regenerate. Collectively, these age‐related changes contribute to a reduction in the speed of electrical impulse transmission through peripheral nerves.
7. The normative reference values established for the Bangladeshi population were compared with those from other population‐based studies [4, 13] that examined asymptomatic healthy controls using standardized techniques. The results of this study are consistent with findings reported in Asian populations (Pakistani, Chinese, Indian, and Malay), while showing notable differences when compared to the American population. No significant differences were observed in motor and sensory conduction velocities within the Asian populations.
8. The reference limits established in Tables 9 and 10 were also compared with the normative datasets of Fong et al. [4] and Preston and Shapiro [14]. Overall, the distal latencies and conduction velocities in this study closely align with Asian values but differ from Western reference parameters. These comparisons reinforce the need for population‐specific normative values for Bangladesh.

Overall, the results have been demonstrated to yield new reference values for the Bangladeshi population and are applicable to assess peripheral nervous system disorders.

## Funding

No funding was received for this manuscript.

## Disclosure

A preprint has previously been published.

## Ethics Statement

The authors confirm that we have read the Journal’s position on issues involved in ethical publication and affirm that this report is consistent with those guidelines.

## Conflicts of Interest

The authors declare no conflicts of interest.

## Data Availability

The original contributions presented in the study are included in the article; further inquiries can be directed to the corresponding author.
